# Predictive and Prognostic Impact of *TP53* Mutations and *MDM2* Promoter Genotype in Primary Breast Cancer Patients Treated with Epirubicin or Paclitaxel

**DOI:** 10.1371/journal.pone.0019249

**Published:** 2011-04-27

**Authors:** Ranjan Chrisanthar, Stian Knappskog, Erik Løkkevik, Gun Anker, Bjørn Østenstad, Steinar Lundgren, Terje Risberg, Ingvil Mjaaland, Gudbrand Skjønsberg, Turid Aas, Ellen Schlichting, Hans E. Fjösne, Arne Nysted, Johan Richard Lillehaug, Per Eystein Lønning

**Affiliations:** 1 Section of Oncology, Institute of Medicine, University of Bergen, Bergen, Norway; 2 Department of Oncology, Haukeland University Hospital, Bergen, Norway; 3 Department of Molecular Biology, University of Bergen, Bergen, Norway; 4 Department of Oncology, Oslo University Hospital, The Norwegian Radium Hospital, Oslo, Norway; 5 Department of Oncology, Oslo University Hospital, Ullevaal, Oslo, Norway; 6 Department of Oncology, St. Olavs University Hospital, Trondheim, Norway; 7 Department of Cancer Research and Molecular Medicine, Norwegian University of Science and Technology, Trondheim, Norway; 8 Department of Oncology, University Hospital of Northern Norway and Institute of Clinical Medicine, University of Tromsø, Tromsø, Norway; 9 Division of Hematology and Oncology, Stavanger University Hospital, Stavanger, Norway; 10 Department of Surgery, Oslo University Hospital, The Norwegian Radium Hospital, Oslo, Norway; 11 Department of Surgery, Haukeland University Hospital, Bergen, Norway; 12 Department of Breast and Endocrine Surgery, Oslo University Hospital, Ullevaal, Oslo, Norway; 13 Department of Surgery, St. Olavs University Hospital, Trondheim, Norway; 14 Department of Surgery, Stavanger University Hospital, Stavanger, Norway; Beth Israel Deaconess Medical Center, United States of America

## Abstract

**Background:**

*TP53* mutations have been associated with resistance to anthracyclines but not to taxanes in breast cancer patients. The *MDM2* promoter single nucleotide polymorphism (SNP) T309G increases *MDM2* activity and may reduce wild-type p53 protein activity. Here, we explored the predictive and prognostic value of *TP53* and *CHEK2* mutation status together with *MDM2* SNP309 genotype in stage III breast cancer patients receiving paclitaxel or epirubicin monotherapy.

**Experimental Design:**

Each patient was randomly assigned to treatment with epirubicin 90 mg/m^2^ (n = 109) or paclitaxel 200 mg/m^2^ (n = 114) every 3rd week as monotherapy for 4–6 cycles. Patients obtaining a suboptimal response on first-line treatment requiring further chemotherapy received the opposite regimen. Time from last patient inclusion to follow-up censoring was 69 months. Each patient had snap-frozen tumor tissue specimens collected prior to commencing chemotherapy.

**Principal Findings:**

While *TP53* and *CHEK2* mutations predicted resistance to epirubicin, *MDM2* status did not. Neither *TP53/CHEK2* mutations nor *MDM2* status was associated with paclitaxel response. Remarkably, *TP53* mutations (p = 0.007) but also *MDM2* 309TG/GG genotype status (p = 0.012) were associated with a poor disease-specific survival among patients having paclitaxel but not patients having epirubicin first-line. The effect of *MDM2* status was observed among individuals harbouring wild-type *TP53* (p = 0.039) but not among individuals with *TP53* mutated tumors (p>0.5).

**Conclusion:**

*TP53* and *CHEK2* mutations were associated with lack of response to epirubicin monotherapy. In contrast, *TP53* mutations and *MDM2* 309G allele status conferred poor disease-specific survival among patients treated with primary paclitaxel but not epirubicin monotherapy.

## Introduction

Anthracyclines and taxanes are the chemotherapeutic agents most frequently used in patients with primary as well as metastatic breast cancer. So far, we have a limited understanding of the mechanisms conferring chemoresistance to both drugs *in vivo*, and we lack suitable predictive factors to select optimal therapy.

Previously, we [1], [2], [3] and others [4] have reported mutations in the *TP53* gene (encoding the tumor suppressor protein p53), with mutations affecting the DNA-binding domains L2/L3 of p53 in particular, to be associated with resistance to anthracyclines in breast cancer patients. While *in vitro* studies have provided conflicting data suggesting a role of p53 to taxane sensitivity [5], [6], the result from the only clinical study available evaluating *TP53* status with respect to paclitaxel sensitivity revealed no correlation [4].

MDM2 (Mouse Double Minute 2 homolog) is an important regulator of p53 and function by suppressing p53 transcriptional activity [7]. Further, *MDM2* amplifications and over-expression have been considered an alternative mechanism of p53 inactivation in several tumor forms [8]. Recently, a single nucleotide polymorphism (SNP) 309 T>G in the *MDM2* intronic promoter (rs = 2279744, referred to as SNP309 in this paper) was found associated with increased *MDM2* mRNA and protein levels [9]. Subsequently, conflicting evidence has linked the SNP 309G variant to enhanced risk of different cancer forms [10]. The predictive value of *TP53* and *CHEK2* mutations on response to chemotherapy in the epirubicin arm of this study has previously been reported [3]. Here, we report the effect of *TP53* and *CHEK2* mutation status on response to paclitaxel. Further, we report the predictive value of *MDM2* SNP309 genotype on response to epirubicin as well as paclitaxel treatment together with long-term follow-up data with respect to disease-specific survival (DSS) for patients in both arms up to a cut-off day 5 year 8 months after the final date of randomization.

## Materials and Methods

### Ethics Considerations

The study protocol was approved by the Regional Ethical Committee (Norwegian Health Region III), including formal Biobank registration in accordance to Norwegian law. The study and protocol is registered under the Norwegian Social Science Data services ((www.nsd/uib/personvern/database/), University of Bergen project no 16297 and Helse Bergen project no 13025). Each patient gave written informed consent.

### Patients

This study enrolled a total of 223 patients with primary stage III breast cancers. Recruitment period was between November 24, 1997 and December 16, 2003. The median age was 51 years (range 25–70). Forty-two patients had a T4 tumor, 177 patients had T3, while three patients had a T2 tumour with concomitant N2 lymph node metastases. One patient was erroneously enrolled with stage II disease. Twenty-four (T3/T4) patients had limited distant metastases (Table 1); these patients were included in the response analysis but omitted from the relapse-free (RFS) and disease-specific survival (DSS) analysis.

**Table 1:** Clinicopathological characteristics and distribution of genotypes analysed.

Clinicopathological factors	Epirubicin cohort	Paclitacel cohort	Total
	n = 109	n = 114	n = 223
**Median age at diagnosis**	51 (range 28–70)	50 (range 25–70)	51(range 25–70)
**Response**			
CR	3 (2.8%)	5 (4.4%)	8 (3.6%)
PR	50 (45.9%)	45 (39.5%)	95 (42.6%)
StD	44 (40.4%)	44 (38.6%)	88 (39.5%)
PD	10 (9.2%)	12 (10.5%)	22 (9.9%)
Missing	2 (1.8)%	8 (7.0%)	10 (4.5%)
**Tumor size**			
T2	2 (1.8%)	1 (0.9%)	3 (1.3%)
T3	92 (84.4%)	85 (74.6%)	177 (79.4%)
T4	14 (12.8%)	28 (24.6%)	42 (18.8%)
T5*	1 (0.9%)		1 (0.4%)
**Lymph node**			
N0	47 (43.1%)	42 (36.0%)	89 (39.9%)
N1	45 (41.3%	57 (50.0%)	102 (45.7%)
N2	15 (13.8%)	15 (13.2%)	30 (13.5%)
N3	1 (0.9%9		1 (0.4%)
N4	1 (0.9%)		1 (0.4)
**Distant metastatis**			
M0	99 (90.8%)	100 (87.7%)	199 (89.2%)
M1	10 (9.2%)	14 (12.3%)	24 (10.8%)
**Oestrogen receptor**			
Positive	59 (54.1%)	66 (57.9%)	125 (56.1%)
Negative	48 (44.0%)	45 (39.5%)	93 (41.7%)
Missing	2 (1.8%)	3 (2.6%)	5 (2.2%)
**Progestron receptor**			
Positive	52 (47.7%)	52 (45.6%)	104 (46.6%
Negative	56 (51.4%)	58 (50.9%)	114 (51.1%)
Missing	1 (0.9%)	4 (3.5%)	5 (2.2%)
**Disease specific dead after 6 years follow-up**	42 (38.5%)	48 (42.1%)	90 (40.4%)
**Median BMI**	26.2 (range 19.3–48.4)	25.4 (range 17.6–41.2)	25.5 (17.6–48.4)
***TP53*** ** mutations**			
All mutations	23 (21.1%)	25 (21.9%)	48 (21.5%)
Mutations in L2/L3 domain	12 (11.0%)	12 (10.5%)	24 (10.8%)
***TP53*** ** LOH**			
WT	72 (67.3%)	51 (45.1%)	123 (55.9%)
LOH	35 (32.7%)	62 (54.9%)	97 (44.1%)
Missing	2	1	3
***TP53*** ** Arg72Pro** (rs1042522)			
GG	77 (70.6%)	77 (67.5%)	154 (69.0%)
GC	26 (23.9%)	27 (23.7%)	53 (23.8%)
CC	6 (5.5%)	10 (8.8%)	16 (7.2%)
***MDM2*** ** SNP309** (rs2279744)			
TT	36 (34.0%)	43 (39.5%)	79 (36.7%)
TG	56 (52.8%)	54 (49.5%	110 (51.2%)
GG	14 (13.2%)	12 (11.0%)	26 (12.1%)
Missing	3	5	8
***CHEK2*** ** mutations**	3 (2.75%)	3 (1.4%)	6 (2.7%)

*One patient was erroneously enrolled with stage II disease.

### Treatment Protocol

This was an open-labelled multicenter study in which patients were randomly allocated to treatment with paclitaxel (n = 114) or epirubicin (n = 109) monotherapy. The reason for randomizing patients between the two arms was not to compare anti-tumor efficacy of the two regimens but to balance patients in the two treatment cohorts with respect to pre-treatment characteristics. The two regimens contained either paclitaxel 200 mg/m^2^ or epirubicin 90 mg/m^2^ administered at 3 week intervals. Treatment was scheduled for four cycles with a possibility for extension based on clinical decisions (see Treatment Protocol uploaded under Supporting Information for details; Protocol S1). Patients obtaining a suboptimal response or progressing on first-line treatment were switched to the opposite regimen in case they were determined to be in need of additional chemotherapy by the treating physician.

All patients harbouring oestrogen receptor (ER) positive tumors (n = 125) were given tamoxifen for 5 years except for postmenopausal patients who were on tamoxifen treatment up to the summer of 2004 and subsequently, were switched to 3 years on treatment with an aromatase inhibitor after completing 2–5 years on tamoxifen treatment (Norwegian Breast Cancer Group Guidelines; www.nbcg.no).The difference in outcome between patients having 5 years on tamoxifen and those switching to an aromatase inhibitor (postmenopausals having received 2 years + on tamoxifen after 2004, but none of the patients having completed 5 years on tamoxifen at that time); is that small that we do not anticipate to see any difference in outcome in a trial of this size.

### Response evaluation

Clinical response was assessed before each treatment cycle. Because the protocol was implemented by October 1997, responses were graded by the UICC system [11]. For consistency, we decided to keep this classification and not change to the recent “RECIST” criteria [12] in the middle of the study period. Thus, responses were classified as; Complete Responder (CR) (complete disappearance of all tumor lesions), Partial Responder (PR) (reduction ≥50% in the sum of all tumor lesion, calculated for each lesion as the product of the largest diameter and the one perpendicular to it), Progressive Disease (PD) (increase in the diameter product of any individual tumor lesion by ≥25%), and Stable Disease (SD) (anything between PR and PD). To analyze for the predictive value of the different parameters, similar to what we conducted in previous studies (2, 17), we compared PD tumors to the combined group of SD/PR/CR tumors [2], [13].

### Tissue sampling

Before commencing chemotherapy, each patient had an incisional tumor biopsy as previously described [1]. All tissue samples were snap-frozen in the operating theatre immediately on removal. For patients switching to the alternative treatment option, a snap-frozen tru-cut biopsy was collected prior to commencing treatment with the second-line drug regimen. Finally, snap-frozen tissue was collected at surgery (mastectomy).

### DNA Purification

Genomic DNA from tumor biopsies was isolated using QIAmp DNA Mini kit (Qiagen, Chatsworth, CA) according to the manufacturer's protocol.

### *TP53* analysis

All *TP53*mutational analyses were performed blinded to clinical data. The complete coding region of *TP53* (NM_000546) was sequenced as previously described [3]. Since normal tissue (required for LOH analysis) was available from 86 patients only, a gene copy number analysis was performed by quantitative PCR using hydrolysis probe-assays (LightCycler 480 system; Roche). Duplex reactions amplifying the genomic area of interest and the Beta-2-microglobulin as an internal reference were performed. (Details regarding primers and PCR conditions are available as Supporting Information; Method S1). Data obtained through the *TP53* specific reactions were normalized by adjusting for Beta-2-microglobulin levels. These normalized values were divided by the corresponding values from a reference sample (pooled DNA from >10 healthy donors). Samples were considered to have reduced copy number if the sample/reference ratio was <0.75, and increased copy number if the ratio was >1.25.

### *CHEK2* analysis

All *CHEK2* mutational analyses were performed blinded to clinical data. The complete coding region of *CHEK2* (NM_007194) was sequenced as previously described [3].

### *MDM2* promoter screening

A region of the *MDM2* (AF_527840) promoter was amplified using the DyNazyme EXT polymerase system (FINNZYMES) according to the manufacturer's instructions with primers MDM2PF- *CGGGAGTTCAGGGTAAAGGT* and MDM2PR- *AGCAAGTCGGTGCTTACCTG* . Thermocycling conditions were an initial step at 94°C, 40 cycles at 94°C for 1 min, 59°C for 30 s and 72°C for 1 min, followed by a final step at 72°C for 7 min. PCR product were sequenced using Big Dye terminator mixture (Applied Biosystems). All sequencing reactions were carried out with the same primers as used for PCR amplification. After an initial step of 5 min denaturation at 94°C, the sequencing reaction was carried out for 40 cycles of 10 s at 94°C, 5 s at 55°C and 4 min at 60. Capillary gel electrophoresis, data collection and sequence analyses were performed on an automated DNA sequencer (ABI 3700).

### Statistical Analysis

All statistical calculations were performed using the SPSS version 15 software package. The differences in the distribution of *TP53* mutations and *MDM2* SNPs among patients revealing a PD and the responders were analyzed using the Fisher's exact test. P-values reported for Fisher's exact tests are given as two-sided and cumulative. Survival analyses were performed by Kaplan-Meier, and subsets of patients were compared using the log-rank test. Patients harbouring distant metastases at the time of diagnosis were excluded from the survival analysis. Deaths for reasons other than breast cancer were treated as censored observations. To explore the effects of several variables and their combined effects on DSS, multivariate Cox regression models were used.

## Results

### *TP53, CHEK2* mutations and *MDM2* status in the patient cohort

Eight patients in the paclitaxel arm and two patients in the epirubicin arm could not be evaluated for treatment response, mainly due to early termination of therapy because of side effects. These patients were included in the RFS and DSS analysis on an intention-to-treat basis. Patients harbouring limited distant metastases in addition to their locally advanced disease at diagnosis (n = 24) were eligible for response assessment but omitted from the RFS and DSS analysis. Thus, 106 and 107 patients were evaluable for treatment response while 100 and 99 patients were included in the overall survival analysis from the paclitaxel and epirubicin arms, respectively.

Clinical stage at diagnosis, objective response rates and major biological findings are summarized in Table 1. *TP53* mutations were identified in 48 (21.5%) of the patients (Table 1); the 23 mutations in the epirubicin cohort has previously been reported [3] but the 25 mutations in the paclitaxel cohort have not been presented earlier (see details regarding individual mutations in Supporting Information Table S1). Out of a total of 48 mutations, 42 were missense, four nonsense and two deletions. Twenty-four of the mutations; 12 in the epirubicin cohort and 12 in the paclitaxel cohort, directly or indirectly affected the L2/L3 domains of the p53 protein critical to DNA binding [14], previously found to predict a poor prognosis in general [15] and drug resistance to anthracyclines and mitomycin in particular [1], [2].

Normal tissue (WBC) from 39 patients was available for germline characterization, revealing two missense mutations to be germline (codon 254 and codon 347; Supporting Information Table S1). Overview regarding the p53 Arg72Pro polymorphism (rs 1042522) and *TP53* LOH status is presented in Table 1.

While three patients in the paclitaxel cohort harboured *CHEK2* mutations (two patients; Arg117Gly, one patients; Ile157Thr) each obtained a PR to treatment. Based on previous characterization [3], these mutants revealed partial agonistic functions.

*MDM2* SNP309 data were available from 215 out of 223 patients (eight patients were not informative). Seventy-nine patients (36.7%) revealed the TT genotype, 110 (51.2%) displayed a TG genotype and 26 (12.1%) were found to hold the GG genotype (Table 1). The polymorphism was shown to be in Hardy-Weinberg equilibrium.

Notably, no pairwise correlation between *TP53* mutation status, the p53 Arg72Pro variant or the *MDM2* SNP309G polymorphism was observed.

### Correlations between *TP53* and *MDM2* status and response to epirubicin or paclitaxel therapy

The influence of *TP53* and *CHEK2* status on response to treatment with epirubicin has been reported previously [3]. Paclitaxel responses in relation to individual mutations are presented in Supporting Information Table S1. While *TP53* mutations, in particular those affecting the L2/L3 domains but also *CHEK2* non-sense mutations, previously shown to be devoid of Chk2 activity [3], predicted lack of response to anthracycline treatment [3], *MDM2* promote*r* genotypes were not associated with response to epirubicin either in the total cohort (n = 107) (p>0.5) or in the subgroup (n = 84) of patients revealing wild-type *TP53* status (p>0.5).

Neither *TP53* mutations in general nor mutations affecting the L2/L3 domains were associated with lack of response to paclitaxel treatment (Table 2).

**Table 2:** Clinical response to paclitaxel in relation to different parameters.

		Clinical response			Statistical significance	
	CR (n = 5)	PR (n = 45)	SD (n = 44)	PD (n = 12)	*P* ^1^	*P* ^2^
*TP53* mutations (n = 25)	3	11	6	5	0.1487	0.4868
*TP53* mutations affecting L2/L3 (n = 12)	2	3	5	2	0.6235	0.6123
*TP53* LOH (n = 53)	2	23	24	7	0.7656	0.7509
*TP53* SNP72 (n = 34)	2	10	18	4	1	0.1464
*CHEK2*mutations (n = 3)		3				
*MDM2* SNP309 (n = 63)	3	28	23	9	0.3526	0.5123
*MDM2* SNP309 (n = 45)*	1	20	19	5	0.2286	0.3805

Clinical response in relation to different parameters. *P* ^1^, with regard to clinical response comparing CR/PR/SD versus PD; *P* ^2^, with regard to clinical response comparing CR/PR versus PD;

*, In the subgroup of the patients revealing wild-type *TP53*.

No association between *TP53* LOH status, the Arg72Pro polymorphism or *MDM2* genotype status and response to either epirubicin or paclitaxel treatment was recorded (p>0.25).

Tumor tissues obtained after paclitaxel treatment (without any addition of epirubicin) was available from five out of 25 patients harbouring *TP53* mutations. Out of these patients, two (Tax260 and Tax106) had SD, while three (Tax086, Tax192 and Tax056) revealed progressive disease. Interestingly, *TP53* mutation status did not change during therapy in any of these patients (Supporting Information Table S1).

### Response to second-line chemotherapy

Forty-one (38.7%) patients obtaining a suboptimal response to paclitaxel and thirty patients (28%) obtaining a suboptimal response to epirubicin received second-line treatment with the opposite regimen. Lack of cross-resistance between anthracyclines and taxanes have been confirmed in multiple studies [16], [17]; thus, potential salvage by second-line therapy may have significant influence on subsequent relapse-free as well as disease-specific survival, masking a potential correlation between response to first-line treatment and RFS and/or DSS. Characteristics of those patients receiving second-line treatment (including response to first- as well as second-line treatment, together with *TP53* mutation status) are depicted in Supporting Information Table S2. Comparing response to epirubicin and paclitaxel administered as second-line versus first-line treatment (Table 3), the frequency of patients obtaining a PD was similar in both settings. However, the likelihood of having a CR/PR on second-line therapy was significantly lower as compared to response to first-line therapy with respect to epirubicin (p = 0.028) as well as to paclitaxel (p = 0.022), consistent with the general observation of lower response rate to second- as compared to first-line therapy in metastatic disease.

**Table 3:** Distribution according to response to chemotherapy as initial and second treatment.

	CR	PR	SD	PD	*P^1^*	*P^2^*
Response to epirubicin as initial treatment	3 (2.8%)	50 (46.7%)	44 (41.1%)	10 (9.3%)		
Response to epirubicin as second treatment	1 (2.4%)	11 (26.8%)	27 (65.9%)	2 (4.9%)	0.512	0.028
Response to paclitaxel as initial treatment	5 (4.7%)	45 (42.5%)	44 (41.5%)	12 (11.3%)		
Response to paclitaxel as second treatment	0 (0.0%)	7 (30.4%)	19 (63.3%)	4 (13.3%)	0.753	0.022

*P* ^1^, with regard to clinical response comparing CR/PR/SD versus PD; *P* ^2^, with regard to clinical response comparing.

CR/PR versus SD/PD.

### *TP53* genotypes and breast cancer survival

**Figure 1:**
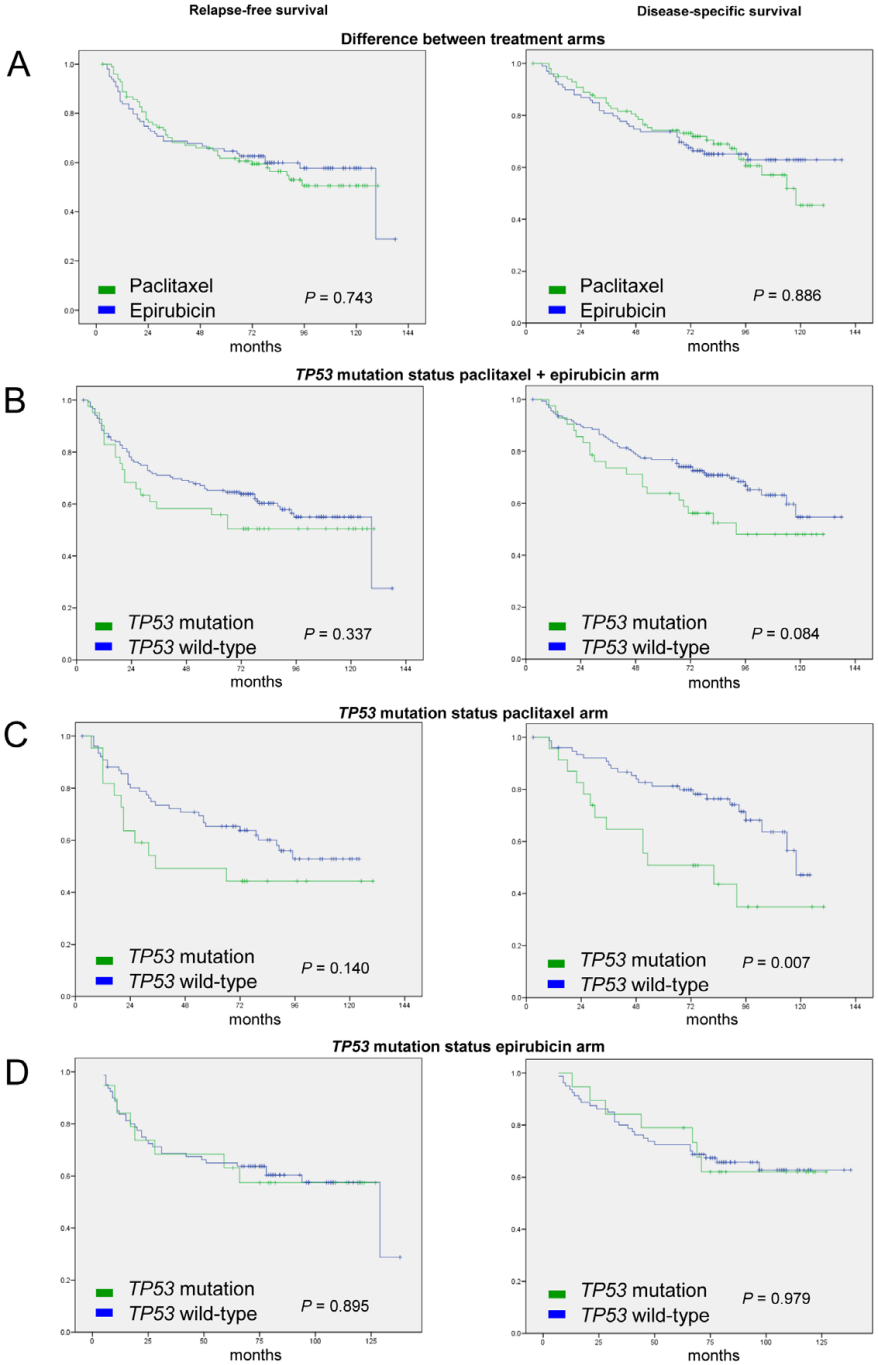
Kaplan-Meier curves of relapse-free (left row) and disease-specific survival (right row). A, Difference between the treatment arms; B, Difference in respect to *TP53* mutation status; C, Difference in respect to *TP53* mutation status in paclitaxel arm; D, Difference in respect to *TP53* mutation status in epirubicin arm.

No difference with respect to RFS or DSS was observed between the two treatment cohorts (p>0.5; Figure 1A). *TP53* mutations were associated with a non-significant trend for reduced DSS (p = 0.084; Figure 1B) but did not influence RFS (p = 0.337) when the two cohorts were analyzed together. The reason for analyzing both cohorts together was to test for a general prognostic effect independent of type of systematic therapy [18]. The difference in DSS was smaller if patients harbouring *TP53* mutations affecting the L2/L3 domain were compared to the combined group of patients revealing *TP53* wild-type status or *TP53* mutations outside the L2/L3 domain (p>0.5). Stratifying patients according to treatment, *TP53* mutations were associated with a significant reduction in DSS (p = 0.007) and a non-significant (p = 0.140) reduction in RFS among patients treated with paclitaxel (Figure 1C) but not among patients receiving epirubicin treatment upfront (Figure 1D). Interestingly, this association for DSS became non-significant when tumors harbouring *TP53* mutations affecting the p53 L2/L3 DNA-binding domains were compared to those with wild-type or *TP53* mutations outside the L2/L3 domains (p = 0.095).

Notably, patients harbouring *TP53* mutations revealed a non-significant trend for an inferior RFS and DSS if treated with paclitaxel as compared to epirubicin as first-line treatment. While patients harbouring wild-type *TP53* tumors did marginally better on paclitaxel (Supporting Information Figure S1), a test for interaction between treatment regimen and *TP53* status with respect to DSS revealed no significant difference (p = 0.165).

To test for potential confounding effects of second-line therapy on DSS, we analysed for DSS excluding all patients having second-line chemotherapy with the alternate drug. Excluding patients having second-line therapy from the DSS analysis had no major effects on outcome (DSS paclitaxel; p = 0.011; epirubicin; p = n.s.).

### *MDM2* genotypes and breast cancer survival

We then investigated the association between the *MDM2* SNP309 genotypes and breast cancer survival. In the first part of the analysis we compared all three groups (309TT, 309TG and 309GG). Due to a small number of patients harbouring the SNP309GG genotype, similar to other studies [19], [20] we compared the combined group of individuals harbouring the 309GG and 309TG genotypes versus 309TT.

**Figure 2:**
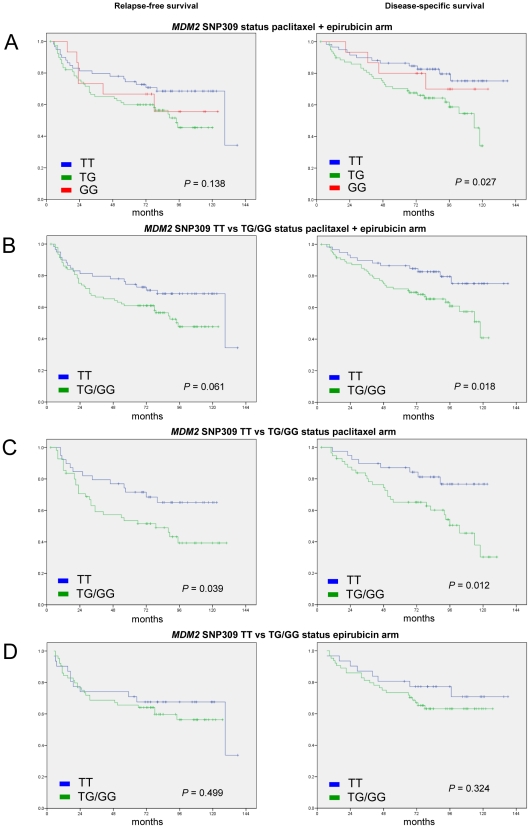
Kaplan-Meier curves of relapse-free (left row) and disease-specific survival (right row). A, Difference in respect to *MDM2 SNP309* status in sub-cohort of the patients harbouring wild-type *TP53*; B, Difference in respect to *MDM2 SNP309* variants when TG and GG were pooled in sub-cohort of the patients harbouring wild-type *TP53*; C, Difference in respect to *MDM2 SNP309* variants when TG and GG were pooled in paclitaxel arm. D; Difference in respect to *MDM2 SNP309* variant when TG and GG were pooled in epirubicin arm.

Taking both patient cohorts together, no difference with respect to RFS (p = 0.261) was observed between *MDM2* SNP309 promoter genotypes. However, a significant correlation was found between *MDM2* SNP309 promoter genotypes and DSS (p = 0.045). This was also the case for the sub-cohort of patients harbouring wild-type *TP53* (RFS; p = 0.138, DSS; p = 0.027, Figure 2A).

Combining patients harbouring the SNP309 TG and GG genotypes from both treatment cohorts, these patients had an inferior outcome as compared to individual harbouring the 309TT genotype (RFS; p = 0.076, DSS; p = 0.010). A similar finding was recorded in the sub-cohort of patients harbouring wild-type *TP53* (RFS; p = 0.061, DSS; p = 0.018; Figure 2B). No effect of *MDM2* SNP309 genotype was recorded in the cohort of patients harbouring *TP53* mutations (RFS; p = 0.815, DSS; p = 0.419). Stratifying patients according to treatment, similar to what was recorded for *TP53* mutation status; we found the *MDM2* SNP309 309TG/GG genotypes to be associated with inferior RFS and DSS in the paclitaxel (Figure 2C) but not in the epirubicin (Figure 2D) cohort. This effect was recorded in the total cohort of paclitaxel-treated patients (RFS; p = 0.039, DSS; p = 0.012, Figure 2C) as well as in the sub-cohort of patients harbouring wild-type *TP53* (RFS; p = 0.086, DSS; p = 0.039).

### Additional potential prognostic factors

Neither *TP53* LOH nor Arg72Pro polymorphism status were associated with RFS or DSS (Supporting Information Table S3). In contrast, ER expression (defined as >10% of cells revealing positive staining) was associated with improved RFS and DSS in both cohorts together (RFS; p = 0.070, DSS; p = 0.005) and in the epirubicin (RFS; p = 0.021, DSS; p = 0.008) but not in the paclitaxel (RFS; p = 0.906, DSS; p = 0.196) arm (Supporting Information Figure S2).

### Multivariate analysis

Factors found to influence DSS in univariate analysis (*TP53* mutation status, *MDM2* genotype SNP309 (TT vs. TG/GG) and ER expression) were evaluated in different models by multivariate Cox regression analysis. The effects were analysed on both arms combined to test for potential treatment interactions and in each arm separately (for listing of parameters into the different models, see Supporting Information Table S3).

Analysing both cohorts together, oestrogen receptor negativity (RR = 2.047, 95% CI = 1.206–3.476, p = 0.008) and *MDM2* SNP309 TG/GG status (RR = 2.039, 95% CI = 1.152–3.610, p = 0.015) both predicted a poor outcome. Excluding oestrogen receptor status from the analysis; *MDM2* SNP309 TG/GG status remained as a poor prognostic factor (RR = 2.034, 95% CI = 1.154–3.585, p = 0.014).

Analysing patients in the paclitaxel arm, only *TP53* mutation status (RR = 2.319, 95% CI = 1.068–5.037, p = 0.033) remained as a negative prognostic factor with a non-significant trend for *MDM2* TG/GG (RR = 2.180, 95% CI = 0.966–4.922, p = 0.061) independent of whether oestrogen receptor status was included in the model.

With respect to the epirubicin arm, oestrogen receptor negativity (RR = 3.381, 95% CI = 1.588–7.198, p = 0.002) remained as a prognostic factor in the multivariate analysis.

## Discussion

Previously, we reported mutations affecting the L2/L3 DNA-binding domain of p53 to be associated with lack of response to weekly anthracycline- as well as mitomycin-containing chemotherapy [1], [2]. More recently, we reported *TP53* L2/L3 mutations, but also non-sense mutations affecting the p53 upstream activator Chk2, to be associated with resistance to primary treatment with epirubicin [3]. Epirubicin acts by DNA intercalation. Treatment with epirubicin will lead to DNA damage in the cell, subsequently activating p53 leading to apoptotic cell death [21], [22], [23], [24], eventually senescence [25].

While *TP53* mutations were associated with anthracycline resistance [2], [3], several tumors revealed chemoresistance despite harbouring wild-type *TP53*. Thus, our recent finding that some of these tumors harboured mutations in the *CHEK2* gene (coding for the Chk2 protein phosphorylating p53 in response to genotoxic stress) indicates defects in the p53 pathway in addition to *TP53* mutations. MDM2 plays a key role regulating p53 function through direct binding, ubiquitination and degradation [26], and MDM2 amplification has been considered an alternative way of inactivating the p53 protein [8]. Due to recent findings of the *MDM2* SNP309T>G polymorphism enhancing *MDM2* transcription and its potential association to increased cancer risk [9], we hypothesized that the *MDM2* SNP309G genotype may be associated with lack of response to an anthracycline regimen. Further, to assess its potential prognostic role, we analyzed its effect on long-term outcome in our two-arm study applying epirubicin versus paclitaxel monotherapy. Notably, *MDM2* genotype was not associated with response to neither epirubicin nor paclitaxel treatment.

Taxanes prevent microtubule degradation, thus leading to cell cycle distortion and apoptosis [27]. Conflicting data from experimental studies have suggested a role for p53 executing apoptosis in response to taxane treatment (5, 6, 21–27). Similar to what has been recorded by others [4], we found *TP53* mutations not to predict resistance to paclitaxel treatment.

Surprisingly, we observed *TP53* mutations to be associated with an inferior long-term DSS among patients receiving paclitaxel but not among patients having epirubicin as first-line therapy. *TP53* mutations are known to be associated with a poor prognosis in breast cancer patients in the absence of adjuvant therapy [28]. One potential explanation why *TP53* mutation status was not associated with prognosis despite predicting resistance to epirubicin in this study could be the fact that patients responding poorly to epirubicin were salvaged by second-line paclitaxel treatment. While excluding patients having second-line treatment from RFS and DSS analysis had marginal effect on the results (data not shown), patients in need for second-line treatment are in general, expected to have a particular poor prognosis; thus, the possibility exist that these patients may have contributed to a different outcome in case they had not receive salvage therapy. Notably, among eight patients having a PD on epirubicin treatment, four had a PR, while two obtained SD on subsequent paclitaxel treatment (Supporting Information Table S2).

While *TP53* mutation status was associated with prognosis among patients having paclitaxel treatment upfront, this effect was found better correlated to *TP53* mutations in total as compared to mutations affecting the L2/L3 domains, contrasting observations from previous studies among patients not treated with chemotherapy [15] as well as patients exposed to anthracycline therapy [2]. Notably, p53 is involved in multiple functions including growth arrest, DNA repair, senescence as well as apoptosis [29]. p53 is phosphorylated or acetylated at multiple sites in response to different stimuli [24] and execute both transcription dependent as well as independent functions [30]. Thus, the possibility exist that p53 may influence prognosis in a different manner among patients treated with paclitaxel as compared to patients having either treatment with an anthracycline or no adjuvant chemotherapy. A prognostic role for *TP53* mutations restricted to the paclitaxel-treated subgroup was indirectly supported by the observation of an inferior prognosis also among patients being either heterozygote or homozygote for the *MDM2* SNP 309G allele. This effect of *MDM2* SNP 309G status on prognosis was observed among patients harbouring wild-type *TP53* in their tumors only, consistent with the biological activity of MDM2 inhibiting p53 activity [9], [31], [32]. Taken together, these observations supports the hypothesis that enhanced *MDM2* activity due to the 309G allele may substitute for *TP53* mutations with the two parameters conferring prognostic impact within similar patient cohorts.

In summary, *TP53* and *CHEK2* mutations previously found associated with lack of primary response does not significantly affect long-term survival among patients receiving epirubicin as first-line treatment. In contrast, *TP53* mutations but also the *MDM2* promoter SNP309 G polymorphism influences long-term survival among patients receiving paclitaxel with large primary breast cancers.

## Supporting Information

Figure S1 **Kaplan-Meier curves of relapse-free and disease-specific survival between treatment arms according to** ***TP53*** **mutation status.**
Kaplan-Meier curves of relapse-free (left row) and disease specific survival (right row). A, Difference between the treatment arms among patients harbouring *TP53* mutations; B, Difference between the treatment arms among patients harbouring wild-type *TP53*.
(TIF)

Figure S2 **Kaplan-Meier curves of relapse-free and disease-specific survival according to oestrogen receptor status.**
Kaplan-Meier curves of relapse-free (left row) and disease-specific survival (right row). A, Difference in survival according to oestrogen receptor status (both treatment cohort together); B, Difference in survival according to oestrogen receptor status (epirubicin arm); C, Difference in survival according to oestrogen receptor status (paclitaxel arm).
(TIF)

Table S1 **Characteristics of** ***TP53*** **mutants found in paclitaxel cohort.**
Nucleotide number; ^1^, The bolded bases indicate the base change; ^2^, Functional predictions derived from a computer model that takes into account the 3D structure of WT and mutant proteins and is trained on the trans activation dataset from Kato et al. Mutations are classified as "functional" or "non-functional". More details here. http://www-p53.iarc.fr/Help.html#StructureClass; T, size or direct of the primary tumour; N, spread to regional lymph nodes; M, distant metastasis; ∧, "F" followed by a number indicates that the patient was free of disease at that number of months of follow-up. "R" followed by a number indicates that the patient was alive at that number of months of follow-up but had suffered a relapse; "A" followed by a number indicates that the patient was alive at that number of months of follow-up. "D" followed by a number indicates that the patient died at that number of months of follow-up; ‡, Characterised as a mutation affecting L2/L3 domain, since it leads to truncation of the protein and will mostly affect L2/L3 domain; AI, Allelic imbalance; * One patient withdrew from the study and was censored after 29 months follow up.
(XLS)

Table S2 **Characteristics of patients switched therapy regime.**
(XLS)

Table S3 **Effect of different factors on disease specific survival by Cox regression univariate and multivariate analysis.**
(XLS)

Protocol S1 **Detailed description of treatment protocol.**
(DOC)

Method S1 ***TP53*** **gene copy number analysis.**
(DOC)
