# Supplementary figures and images for: Predictive and Prognostic Impact of TP53 Mutations and MDM2 Promoter Genotype in Primary Breast Cancer Patients Treated with Epirubicin or Paclitaxel
Source: PLoS One. 2011 Apr 27;6(4):e19249. doi: 10.1371/journal.pone.0019249 (PMC3083424; doi:10.1371/journal.pone.0019249)

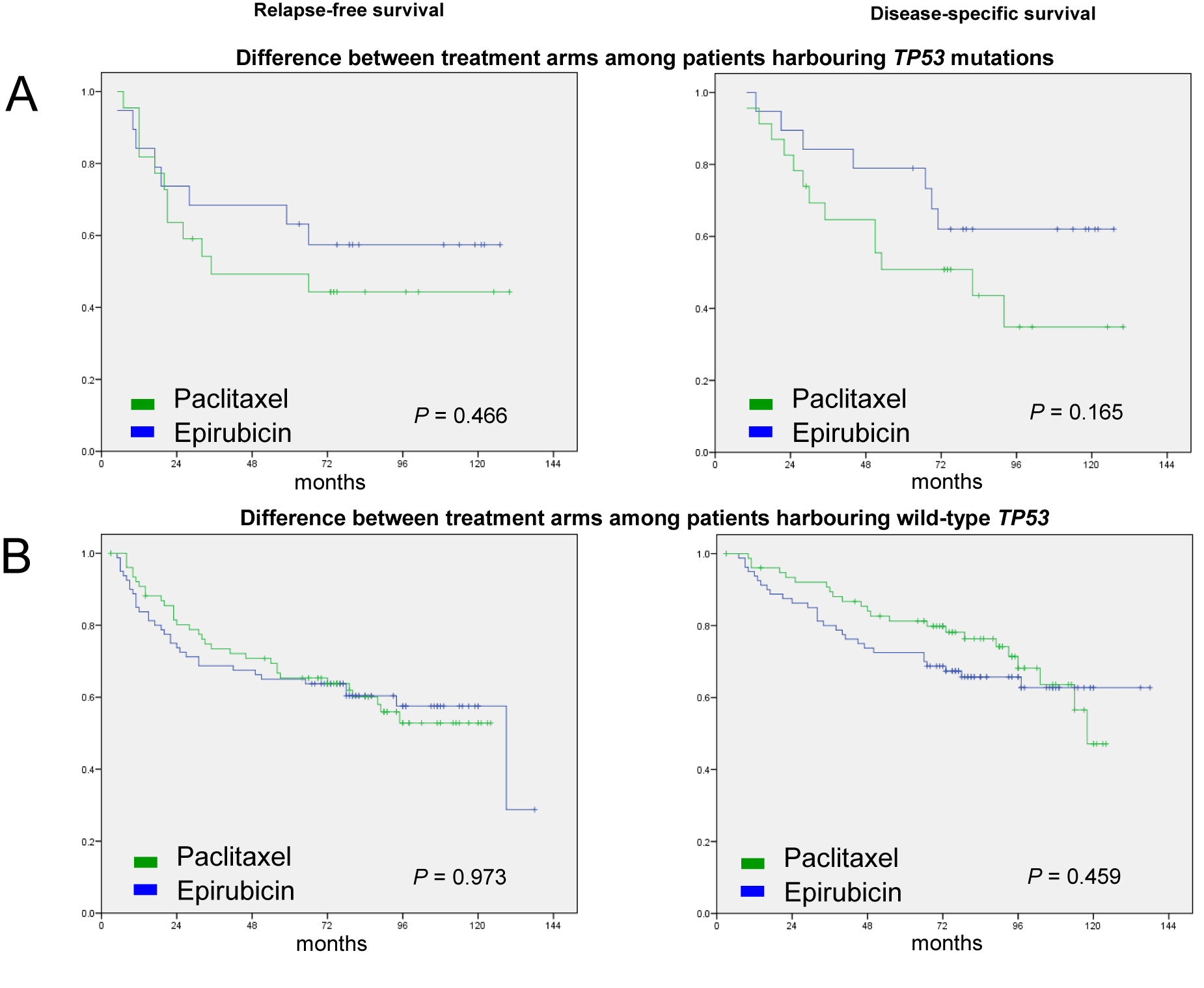

Supplement: Figure S1 — Kaplan-Meier curves of relapse-free and disease-specific survival between treatment arms according to TP53 mutation status. Kaplan-Meier curves of relapse-free (left row) and disease specific survival (right row). A, Difference between the treatment arms among patients harbouring TP53 mutations; B, Difference between the treatment arms among patients harbouring wild-type TP53. (TIF) [file pone.0019249.s001.tif]

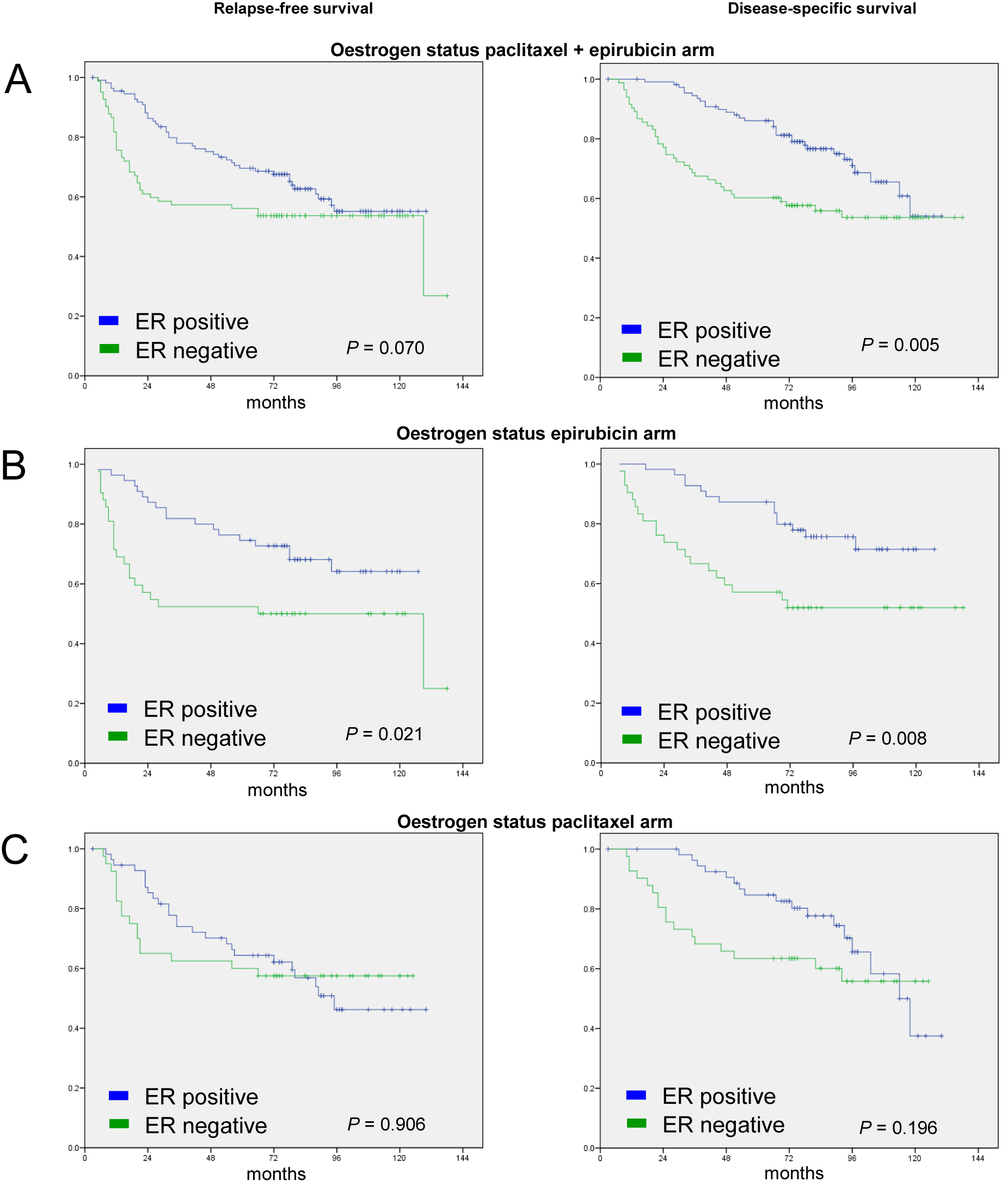

Supplement: Figure S2 — Kaplan-Meier curves of relapse-free and disease-specific survival according to oestrogen receptor status. Kaplan-Meier curves of relapse-free (left row) and disease-specific survival (right row). A, Difference in survival according to oestrogen receptor status (both treatment cohort together); B, Difference in survival according to oestrogen receptor status (epirubicin arm); C, Difference in survival according to oestrogen receptor status (paclitaxel arm). (TIF) [file pone.0019249.s002.tif]
