# Supplementary material for: Predictive and Prognostic Impact of TP53 Mutations and MDM2 Promoter Genotype in Primary Breast Cancer Patients Treated with Epirubicin or Paclitaxel
Source: PLoS One. 2011 Apr 27;6(4):e19249. doi: 10.1371/journal.pone.0019249 (PMC3083424; doi:10.1371/journal.pone.0019249)
Supplement: Protocol S1 — Detailed description of treatment protocol. (DOC) [file pone.0019249.s006.doc]

**Treatment Protocol**

**Evaluation of epi-adriamycin versus paclitaxel for primary medical treatment ("neo-adjuvant chemotherapy") in patients with locally advanced (stage III, T3/4 and/or N_2_) non-inflammatory breast cancer with or without limited distant metastases; phase II study.**

**Including amendments of October 24, 1997 (I), February 16, 1998 (II) and June 23, 1999 (III).**

**June 23; revisions done to section “Clinical assessment”, “Biochemistry” and “Investigations on bone marrow aspirates….”; all changes in bold.**

**Senior Chairman** Per E. Lønning

Professor in Oncology, Department of Oncology,

Haukeland University Hospital, Bergen, Norway

**Background**

Locally advanced breast cancer is a serious condition offering a therapeutic challenge to the surgeon as well as to the oncologist. Official records report stage III breast cancer (tumour status T3/4 and/or N2 without distant metastases) to account for about 6% of all breast cancers annually diagnosed in Norway. However, our experience from Haukeland University Hospital based on a recently conducted prospective study suggests the figures to be in the range of 12-14%, probably due to mis-classification of small T3 tumours in the T2 category by many surgeons 1 In addition, some patients may have locally advanced breast cancer as part of stage IV disease.

The demography of patients suffering from locally advanced breast cancer seems to vary substantially between different countries. While several authors have reported a median age of about 50 years in patients with stage III breast cancer, in Bergen we found a median age of 64 years among patients in our recent study conducted at Haukeland University Hospital 2 In this study we did not enroll patients with inflammatory carcinomas (which often occurs at an earlier age). However, it is clear that a substantial number of patients presenting with locally advanced breast cancers are senior patients, and thus may have a limited tolerance to toxic chemotherapy.

While the percentage of patients with stage III breast cancer staying disease-free 5 years after primary treatment varies between different studies depending on patient characteristics (e.g. tumour size, T3 versus T4 or inflammatory tumours), figures in the range of 20-30% have been reported 3,4.. Apart from risk of developing systemic metastases, patients with stage III breast cancer also suffer a high risk of achieving locoregional relapses 5-7.

There is no general consensus considering how to treat locally advanced breast cancer; little information from randomized studies is currently available. However, the general opinion is that these patients are in need of multimodal treatment, as radiotherapy or surgery alone or in concert do not provide satisfactory results 6,8-10. During the recent years, a popular approach has been to administer primary systemic treatment, so-called "neo-adjuvant" therapy.

Administration of systemic treatment as first line therapy provides several advantages. First, it offers the possibility to "down-stage" the tumour prior to surgery. While conventionally this was done with use of radiotherapy, surgery following radiotherapy is associated with an increased risk of local complications (infections, delayed wound healing) 11. Second, primary systemic treatment provides an opportunity to directly assess the clinical effect of therapy on the primary tumour. Postulating that drug sensitivity to be similar in the primary tumour and micro-metastases, primary systemic treatment offers the possibility to test directly for chemosensitivity before application of long term adjuvant systemic treatment. Finally, primary systemic treatment implicates early systemic treatment which probably may increase the chance of eradicating micrometastases.

While there are no data from randomized studies comparing the effect of primary systemic therapy to other treatment options in stage III breast cancer patients, the numerous phase II reports on use of primary systemic treatment published during the recent years allow some conclusions to be drawn:

1) While there is no consensus on which drug regimen should be applied, a general impression is that most drug regimens used for neoadjuvant treatment seem to cause a somewhat higher response rate in this setting compared to what is achieved when the same regimens are used for treatment of metastatic breast cancer 12-15.

2) So far we lack definite data whether neoadjuvant treatment for stage III breast cancer may influence long term risk for relapse and breast cancer death. However, it is a clear impression that primary chemotherapy given in concert with appropriate local therapy may reduce the risk of a local relapse in stage III patients 1. Due to the high risk of uncontrolled, locoregional relapses in these patients, a significant reduction in the incidence of such relapses justifies use of primary systemic treatment independent of whether it may influence the risk of distant relapses or not.

**Rationale for protocol**

As stated above, there is no general agreement which drug regimen may be the optimal one for treatment of locally advanced breast cancer. Traditionally, chemotherapy for breast cancer has been applied as multidrug treatment using 2-4 different drugs in concert. However, considering drugs shown to be highly effective in breast cancer on their own (like anthracyclines), there is little evidence suggesting these drugs to act more effective in combined regimens compared to single drug treatment provided the drugs are administered in "equipotent" doses (≈ regimens that produce a similar toxicity).

In a previous study we used doxorubicin as weekly monotherapy (14 mg/m^2^ per week) in patients with stage III breast cancer. This drug was well tolerated in senior patients even up to an age of >80 years 1. Forty-nine percent of our patients obtained a "partial response" to therapy, which is somewhat lower than what has been reported for more toxic drug regimens used in the primary setting but better than what has been achieved with weekly doxorubicin 20 mg in metastatic breast cancer 16. While tumour specimens from this study are currently being analyzed for biochemical parameters of relevance to chemoresistance, some interesting results have already emerged, in as much as certain mutations in the *TP53* gene were found to be associated with primary resistance to doxorubin therapy 2.

Over the recent years there has been an increasing interest in exploring more aggressive chemotherapy for breast cancer. A reasonable approach would be to explore anthracycline treatment for locally advanced breast cancer further but with a higher dose intensity compared to what we used in our previous study. There are several reasons advocating use of anthracyclines albeit in a different dose regimen in this setting without selectively excluding patients with certain *TP53* mutations. First, *in vitro* experiments have shown different chemotherapeutic agents to induce apoptosis in a p53 independent matter when applied in doses higher than the ones required to induce p53-dependent apoptosis 17. In addition, recent studies have shown "low dose chemotherapeutic regimens" applied weekly to work better in patients with low grade tumours 18,19, which contrast observations made in relation to "conventional" chemotherapy with 3-4 weekly regimens. This may suggest that different antitumour mechanisms of action could be involved.

Noteworthy, our previous regimen (doxorubicin dose of 14 mg/m^2^, providing a mean dose of about 23-24 mg in most patients) was found to be associated with more side effects compared to what we previously experienced with fixed doses of doxorubicin 20 mg weekly in metastatic breast cancer 16. In particular, there was a significant increase in the number of patients temporarily requiring a wig due to alopecia. Accordingly, if we wish to escalate the dose of anthracyclines further, there may be little benefit in administering the drug at weekly compared to 3-weekly intervals. Assuming other side effects, like nausea, to be increased by dose escalation, it may be more tolerable to the patient to have the drug administered at 3-weekly intervals. This view is supported by others 20 who found doxorubicin given as 75 mg/m^2^ every third week to be better tolerated compared to doxorubicin administered weekly at a dose of 25 mg/m^2^.

Primary systemic treatment of locally advanced breast cancer offers an unique opportunity to explore possible mechanisms of resistance to chemotherapy *in vivo*. Chemoresistance is the main obstacle to a successful treatment of breast cancer patients suffering from locally advanced or metastatic disease. In our previous study, all patients underwent a surgical biopsy for tissue sampling for research purposes prior to surgery. The hypothesis was to explore possible biochemical parameters predicting for a response or resistance to anthracycline therapy. The preliminary findings of this study merit consideration, as they illustrate how the findings from such studies may extend our knowledge about the mechanisms of chemoresistance. In this study, we found primary resistance to doxorubicin therapy given as the regimen outlined above to be associated with particular mutations in the *TP53* gene.

Paclitaxel belongs to the taxanes, an interesting new group of drugs shown to be effective given as monotherapy in breast cancer 21. A most important finding is recent observations suggesting *TP53* mutations not to confer resistance to taxanes. Indeed, *TP53* mutations were shown to sensitise cancer cells to the toxic effects of taxanes in vitro 22. Although the number of observations so far is limited, results from clinical studies have revealed lack of complete cross- resistance to taxanes and anthracyclines in patients with advanced breast cancer 21. These findings provide a rationale for evaluating treatment with taxanes and anthracyclines in primary breast cancer in a cross-over design.

Based on the background provided above, this protocol is designed to evaluate epi-adriamycin versus paclitaxel monotherapy as first line treatment in patients with stage III breast cancer. Patients responding to treatment (CR or PR) will receive local treatment while those showing a non-satisfactory response (MC, SD or PD) to primary treatment with one regimen will be crossed over to the other treatment regimen.

**Aims of the study**

**Primary aims**:

- to evaluate the clinical effects of epi-adriamycin monotherapy and paclitaxel monotherapy, both drugs administered in optimal doses, and to explore cross-resistance towards the two regimens in patients with locally advanced breast cancer.

- to explore parameters predicting for chemoresistance to epi-adriamycin and paclitaxel monotherapy in primary breast carcinomas.

**Secondary aim:**

- to evaluate the incidence of bone marrow metastases in patients with locally advanced breast cancer and to evaluate the prognostic impact of this parameter as well as other tumour parameters on relapse-free survival and overall survival in this patient group.

**Design**

Randomized, open design with cross-over in non-responders.

**Drug regimens**

Dose schedules: The dose of epi-adriamycin is selected partly on the basis of results published in a recent Danish study 23 while the dose of paclitaxel is selected on basis of a general overview of the literature.

Epi-adriamycin: 90 mg/m^2^ every third week.

Paclitaxel. 200 mg/m^2^ every third week.

**Patients.**

**Inclusion criteria.**

- women aged ≥ 18 years and ≤70 years of age with locally advanced (stage III: T3/4 and/or N2) non-inflammatory tumours in whom primary systemic treatment is indicated.

**Exclusion criteria.**

- impaired renal function (defined as creatinine ≥ 1.5 times the upper normal limit)

- ALAT ≥ 1.5 times upper normal limit (except in patients with solitary liver metastasis accepted for the study)

- total serum Ca^2+^ ≥ 10% above upper normal limit

- previous malignancies, except for in situ cancer of the uterine cervix, basal cell skin cancer or spinocellular skin cancer without any evidence of relapse for ≥ 5 years

- multiple distant metastases (patients with limited distant metastases, like limited locoregional (skin, subcutaneous or lymph nodes on the truncus/supracalvicular fossa or contralateral axilla), limited skeletal metastases with ALP ≤ 2 times its upper normal limit or solitary lung or liver metastases for whom, in the opinion of the investigator, their local tumour constitutes their major therapeutic challenge, may be enrolled in the protocol provided they are candidates for surgical treatment of their local tumour after chemotherapy)

- inflammatory carcinoma

- previous allergic reactions to chromophore

**Diagnosis and treatment**

Notice that all time intervals regarding on treatment and in the follow-up setting refer to time from the day of randomisation (recorded on form 5 in the patient registration form book).

All patients eligible for this trial should be recorded by full name and date of birth on the patient log form kept by the responsible investigator in each individual centre. This log will not be made available to the trial centre. The reason for it is two-fold: a) to allow the responsible investigator in each centre to identify each patient enrolled (as they are coded in the registration form) and b) to allow the investigators by the end of recruitment to account for the total number of eligible patients that, for any reason refuse to participate in the study.

**Staging prior to treatment**

Prior to having a surgical biopsy, each patient will have a bilateral mammography. Whenever possible, patients will have their breast tumour (and / or, in case of a large axillary lump, the axillary tumour) assessed by a NMR scan (not mandatory). In addition, patients will be staged for distant metastases. The investigations should include:

- chest X-ray

- ECK

- X-ray of the pelvic area and lumbar spine (not mandatory if bone scintigram is normal)

- liver ultrasound

- bone scintigram

"Hot spots" on the scintigrams will require X-ray examinations of these areas.

In case staging does not provide evidence of distant metastasis, there is no need to repeat any of these investigations during the study period.

**Clinical assessment**

Clinical assessment of tumour response will be by comparison of caliper measurements made by the same clinician (i) just prior to commencing chemotherapy, (Notably, the time interval between surgical biopsy and pre-treatment measurement of tumour size should be ≥ 14 days to avoid any confounding influence of post-surgical bleeding or oedema) and (ii) before each cycle of chemotherapy (4 cycles) and 3 weeks after the forth cycle (see later). **Considering before-treatment measurement, for practical reasons this may be done prior to the surgical biopsy, provided that the largest diameter of the tumour is ≥ 5 cm and the perpendicular diameter ≥4 cm.**

Patients whose tumour response is classified as "progressive disease" at any stage will be crossed over to the alternate chemotherapy regimen.

Similar, patients with "stable disease" on first line chemotherapy when attending the clinic for their forth chemotherapy cycle will not receive this cycle but instead be crossed over to the alternative treatment option They should receive their first cycle of second line treatment within 8 days.

Patients with a "minimal change" response on first line chemotherapy when attending the clinic for their forth chemotherapy cycle should receive this 4th cycle with first line chemotherapy. When examined 3 weeks later (before eventually receiving a 5th cycle), in case of no further significant reduction (compared to 3 weeks before) the patient should not receive her 5th cycle with first-line therapy but instead will be crossed over to the alternative treatment option and should receive second line treatment within 8 days. In case of a significant reduction in tumour size after cycle 4 (but still not achievement of a "partial response", the patient may still receive further treatment with first line chemotherapy (cycle 5 and, eventually further cycles) based on the clinical judgement by the responsible physician (like for patients with "partial responses", see later)."

Patients with a "partial" or "complete" response following 4 cycles of chemotherapy will, in case they are technically operable, have surgery to the breast within 14 days after receiving their forth treatment cycle. However, patients experiencing a "partial response" but with tumours still not operable (large T4 tumours etc.) may receive up to a total of 8 cycles of chemotherapy using the same first line chemotherapy regimen to achieve optimal dawn-staging prior to local therapy. In this case, tumour size should be assessed prior to each chemotherapy cycle. Treatment should be interrupted if evidence of "progressive disease" (increase of ≥25% in the size of any lesion evaluated as the product of its largest and perpendicular diameter) or if tumour stays stable (reduction in size of total tumour burden evaluated as the sum of the perpendicular products) by < 25% 3 weeks after the third cycle of chemotherapy (similar practice for second line therapy).

**Biochemistry**

**Plasma samples**

Fasting blood samples will be collected in the mornings (between 07.00 and 10.00) on the following days:

1) the incisional biopsy

2) the day after this

3) the day of the Tru-cut biopsies

4) three weeks after the third chemotherapy cycle (before the forth cycle or just prior to changing therapy)

In case of cross-over to second line chemotherapy:

5) the day of the third cycle of second line chemotherapy

In addition, on one occasion a blood sample (10 ml) is collected in an EDTA-containing vial. This should be submitted to the Laboratory of Medical Genetics (Professor Børresen-Dales laboratory) for LOH analysis. Practically, this sample may be obtained at the same time as the first bone marrow aspiration (or later, in case it is forgotten) and submitted together with the other samples to dr. Kvalheim who will see that it goes to its right address.

**Investigations on tumour tissue**

**Tissue collection and handling**

**(a) Before commencing on treatment:**

A surgical incisional biopsy will be performed. Tissue will be obtained for routine pathological/anatomical diagnosis performed in the individual center and for immunohistochemical and/or biochemical definition of ER status (optional; preferably, both methods should be performed but immunohistochemical staining only is mandatory). In addition, a paraffin-embedded tumour specimen should be prepared and submitted to the trial center. (Note: we prefer to have all these samples from each patient submitted together; see Logistic manual.) Fresh tissue (about 1 gram in all) will be obtained for research purposes. This should be chopped into individual samples (6 samples, each consisting of 100-150 mg of tissue), snap-frozen in nitrogen in the theatre and stored in individual vials in liquid nitrogen.

**(b) The day after first course of chemotherapy:**

One or, if possible, 2 Tru-cut needle biopsies (needle size to be determined) will be taken and the tissue will be fixed in formalin and embedded in paraffin for research purposes (immunohistochemical investigations on apoptosis). This should be submitted to the trial center as outlined above.

**(c) Following 3 - 4 cycles of chemotherapy:**

Patients with shrinkage of their tumour sufficient to render it operable will subsequently have the tumour surgically removed following 4 cycles of chemotherapy. Research samples will be collected similar to those taken prior to treatment. One piece should be paraffin-embedded and (preferably) 6 pieces snap-frozen in nitrogen.

Patients not responding to treatment, will be given alternative therapy according to the protocol. In those patients not suitable for surgery, a second incisional biopsy or at least 2 Tru-cut biopsies should be obtained prior to any further treatment (following 4 cycles of chemotherapy or earlier in case of "progressive disease"). In case of Tru-cut biopsies, part of the samples should be paraffin-embedded while the rest of the samples should be snap-frozen in nitrogen. Notice a difference in practice compared to b, in as much as we here request both paraffin-embedded and snap-frozen tissue.

**Parameters to be measured in the tumour specimens**

The following parameters are to be determined (a) before, (b) the day after first course of chemotherapy, (c) after 3 - 4 cycles of first line chemotherapy and (in case of second line chemotherapy or extended first line therapy) by the end of chemotherapy.

**Pathology**

The tumours will be independently classified and graded by two pathologists accarding to standard criteria as previously used 2. Associations between pathological features and outcome (clinical response, recurrence and survival) will be examined.

**Primary end-points**

**- parameters of possible relevance to drug resistance:**

- P53 status (evaluated as genetic mutations and by immunohistochemical expression of protein)

- Bcl-2, p21, Bax

- P-glycoprotein (immunohistochemically)

- other possible parameters of relevance evaluated by immunohistochemistry (GSTs, Topoisomerases and so on)

**- apoptosis:**

- in situ labeling

**Secondary end-points**

- expression of growth factors and growth factor receptors (immunohistochemically)

- evaluation of bone metastases in bone marrow aspirates.

**Investigations on bone marrow aspirates and assessment of tumour cells in peripheral blood samples**

Bilateral christa aspirates and peripheral blood samples are taken on 3 occasions: 1) prior to commencement of chemotherapy, 2) on the day of mastectomy when the patient is under general anesthesia and 3) 12 ± 2 months after the day of randomization. Samples should be collected and handled as outlined in protocol amendment. Important: Notice that sampling on the day of mastectomy should allways be performed prior to any surgical procedure, as this may cause shedding of tumour cells and contaminate the speciemn**. Regarding aspirations on other occasions (prior to therapy and after 12 months), in case it is performed after a biopsy, the time interval should be ≥48 hours.** Also notice that these aspirations, similar to the ones obtained prior to treatment and after 1 year, should be obtained from the bilateral christa iliaca (back) to have a similar procedure on each occasion. For all aspirations to be performed without general anaesthesia, a combination of a strong pain-killer (morphine or fentalyl) by the parentheral route and local anaesthesia is recommended. **Notice information in Logistic Manual regarding how to mark the bone marrow samples.**

**Number of patients to be enrolled:**

The aim of this study is not to detect a statistically significant difference in response rate to the two drug regimens but to evaluate possible parameters predicting primary resistance to each drug regimen and to explore clinically cross-resistance to the drug regimens in patients having an inadequate response to first line treatment.

Currently, we have little data available to predict percentage cross-resistance (or lack of such) between the two treatment options. A major goal is to evaluate our hypothesis based on previous findings that patients harboring certain *TP53* mutations in their tumours (those affecting the L2/L3 domain of the p53 protein molecule) have poor response to primary chemotherapy. Based on the assumption that 15% of patients express such p53 mutations and 40% of patients with such mutations to have PD on first line therapy compared to 4% of patients without such mutations, an alpha- and beta-value of 5% and 0.8 would require at least 42 patients to be enrolled in each arm. This calculation is based on a chi-square model without using the Yates correction. Including this factor, we need to increase the number of patients to 60 in each arm. Similar calculations (chi-square with use of Yates correction) give a number of patients of 81 using an alpha value of 0.05 and a beta of 0.9. Using an alpha value of 0.01, the number of patients required is 90 and 114 respectively using a beta-value of 0.8 and 0.9 respectively.

Assuming 5-10% of patients enrolled to be ineligible, a total number of 100 patients should be enrolled in each arm.

**Chemotherapy.**

Each patient is randomly allocated to primary treatment with either epi-adriamycin or paclitaxel in doses as outlined every third week. Each drug is administered as an i.v. infusion (epi-adriamycin for about half an hour, paclitaxel for 3 hour).

**The drug regimens are:**

Regimen A: Epi-adriamycin (Farmorubicin): 90 mg/m^2^ every third week.

Regimen B: Paclitaxel (Taxol): 200 mg/m^2^ every third week.

Chemotherapy is administered at a full dose schedule as long as WBC ≥ 3.0 and platelets ≥ 50. WBC and platelets should be determined on day 8. In case WBC ≤1.5 or platelets ≤50, the parameters should be determined every other day until nadir.

**Dose reductions should be performed as follows:**

For both regimens, a suppression of WBC ≤ 1.0 for at least 3 days requires a dose reduction of 10%. The patient should then stay on this dose (or, if required, further reduction) for the rest of the treatment cycles.

If WBC ≤ 3.0 or platelets ≤50, postpone treatment for up to one week to reach adequate levels. If values are still too low for treatment, the patient should terminate chemotherapy.

Due to side effects caused by paclitaxel treatment, each patient should receive pre-medication as follows:

- dexamethasone 20 mg p.o. 12 and 6 hours prior to paclitaxel infusion

- deksklorfeniramin 5 mg i.v. 30-60 minutes prior to infusion

- rantidin 50 mg i.v. 30-60 minutes prior to infusion

Dexamethasone plays an important role for anti-emetic treatment in relation to anthracycline therapy. Because dexamethasone given in the doses outlined above in theory could have anti-tumour activities, dexamethasone should be administered also to patients receiving epi-adriamycin therapy. In addition, these patients should be given metoclopramid and, in case this regimen does not palliate emesis, 5HT3-receptor-antagonists (but should still receive dexamethasone).

**Surgery**

Patients should (whenever possible) be treated with a simple mastectomy (breast conservation surgery is not recommended). Routine axillary dissection is not used for staging purposes. However, all patients who at any stage have palpable lymph nodes recorded should be surgically explored and any enlarged glands should be removed for therapeutic reasons.

**Radiotherapy**

All patients treated with mastectomy should receive radiotherapy to the axilla and chest wall (50 Gy) and to the supraclavicular fossa (48 or 50 Gy; dose optional, depending on current practice in individual centres). In case of tumour infiltration at the edge of the specimen, a booster (up to 10 Gy) is applied to the mammary bed in accordance with general practice.

**Endocrine treatment**

All patients expressing receptor positive tumours (biochemical ER and/or PgR ≥ 10 fmol or immunohistochemical staining for either of the receptors in ≥10% of the cells) should receive tamoxifen 20 mg o.d. for 5 years. Notably, tamoxifen treatment should not be implemented until after surgery (or, in case the patient was found unfit for surgery, the patient may commence on tamoxifen at the time of initiating radiotherapy and after obtaining relevant Tru-cut biopsies).

While a recent study 24 reported a benefit of adding another 3 cycles of late chemotherapy following 3 months of adjuvant chemotherapy to pre-menopausal women, we do not know whether similar effects relate to postmenopausals and patients treated for stage III disease. Our previous experience at Haukeland University Hospital suggest that most patients treated with pre-surgical chemotherapy followed by surgery and radiotherapy will be reluctant to have further chemotherapy. Thus, we do not recommend any further chemotherapy after surgery and radiotherapy.

**References**

1. Aas T, Varhaug JE, Kolnes J, et al: Primærbehandling av lokalavansert brystkreft med bruk av neoadjuvant kjemoterapi. Tidsskr. Nor. Laegeforen. 114:668-670, 1994

2. Aas T, Børresen A-L, Geisler S, et al: Specific P53 mutations are associated with *de*

3. Rubens RD: The management of locally advanced breast cancer. Br. J. Cancer 65:145-147, 1992

4. McGuire WL, Abeloff MD, Hortobagyi GN, et al: Treatment of stage III breast cancer. Breast Cancer Res. Treat. 13:225-235, 1989

5. Montague ED, Fletcher GH: The need for every modality treatment to prevent catastrophic local and regional failures in advanced breast cancer. Int. J. Radiat. Oncol. Biol. Phys. 9:1625-1630, 1983

6. Bruckman JE, Harris JR, Levene MB, et al: Results of treating stage III carcinoma of the breast by primary radiation therapy. Cancer 43:985-993, 1979

7. Spanos Jr. WJ, Montague ED, Fletcher GH: Late complications of radiation only for advanced breast cancer. Int. J. Radiat. Oncol. Biol. Phys. 6:1473-1476, 1980

8. Haagensen CD: Diseases of the breast. Philadelphia, Saunders, 1989

9. Amalric R, Santamaria F, Robert F, et al: Radiation therapy with or without primary limited surgery for operable breast cancer. Cancer 49:30-34, 1982

10. Gröhn P, Heinonen E, Klefström P, et al: Adjuvant postoperative radiotherapy, chemotherapy, and immunotherapy in stage III breast cancer. Cancer 54:670-674, 1984

11. Sauter ER, Eisenberg BL, Hoffman JP, et al: Postmastectomy morbidity after combination preoperative irradiation and chemotherapy for locally advanced breast cancer. World J. Surg. 17:237-242, 1993

12. Swain SM, Sorace RA, Bagley CS, et al: Neoadjuvant chemotherapy in the combined modality approach of locally advanced nonmetastatic breast cancer. Cancer Res. 47:3889-3894, 1987

13. Piccart MJ, de Valeriola D, Paridaens R, et al: Six-year results of a multimodality treatment strategy for locally advanced breast cancer. Cancer 62:2501-2506, 1988

14. Jacquillat C, Baillet F, Weil M, et al: Results of a conservative treatment combining induction (neoadjuvant) and consolidation chemotherapy, hormontherapy, and external and interstitial irradiation in 98 patients with locally advanced breast cancer (IIIA-IIIB). Cancer 61:1977-1982, 1988

15. Hortobagyi GN, Ames FC, Buzdar AU, et al: Management of stage III primary breast cancer with primary chemotherapy, surgery, and radiation therapy. Cancer 62:2507-2516, 1988

16. Gundersen S, Kvinnsland S, Klepp O, et al: Weekly adriamycin versus VAC in advanced breast cancer. A randomized trial. Eur. J. Cancer Clin. Oncol. 22:1431-1434, 1986

17. Lowe SW, Ruley HE, Jacks T, et al: p53-dependent apoptosis modulates the cytotoxicity of anticancer agents. Cell 74:957-967, 1993

18. Aas T, Geisler S, Paulsen T, et al: Primary systemic treatment with weekly doxorubicin monotherapy in women with locally advanced breast cancer; clinical experience and parameters predicting outcome. Acta Oncol. 35:5-8, 1996

19. Sjöström J, Blomqvist C: Predictive factors for response to cytotoxic treatment in advanced breast cancer. A review. Acta Oncol. , In press

20. Richards MA, Hopwood P, Ramirez AJ, et al: Doxorubicin in advanced breast cancer: Influence of schedule on response, survival and quality of life. Eur. J. Cancer 28A:1023-1028, 1992

21. Lønning PE: Paclitaxel and Docetaxel. Innovation, But at what cost? PharmacoEconomics 8:1-4, 1995

22. Wahl AF, Donaldson KL, Fairchild C, et al: Loss of normal p53 function confers sensitization to taxol by increasing G2/M arrest and apoptosis. Nature Med. 2:72-79, 1996

23. Bastholt L, Dalmark M, Gjedde SB, et al: Dose-response relationship of epirubicin in the treatment of postmenopausal patients with metastatic breast cancer: A randomized study of epirubicin at four different dose levels performed by the Danish Breast Cancer Cooperative Group. J. Clin. Oncol. 14:1146-1155, 1996

24. International breast Cancer Group; Duration and reintrductn f adjuvant chemotherapy for node-positive remenopausal breast cancer patients. J. Clin. Oncol. 6: 1885-1894, 1996.
