# Supplementary material for: Predictive and Prognostic Impact of TP53 Mutations and MDM2 Promoter Genotype in Primary Breast Cancer Patients Treated with Epirubicin or Paclitaxel
Source: PLoS One. 2011 Apr 27;6(4):e19249. doi: 10.1371/journal.pone.0019249 (PMC3083424; doi:10.1371/journal.pone.0019249)
Supplement: Method S1 — TP53 gene copy number analysis. (DOC) [file pone.0019249.s007.doc]

Method S1. TP53 gene copy number analysis

Gene copy number of *TP53* determined using hydrolysis probe-assays on the LightCycler 480 system (Roche). Duplex reactions amplifying *TP53* and *B2M* (Beta-2-microglobulin), as an internal reference in the same reaction well, were performed for two distinct area of *TP53* in a 20 µl reaction solution containing 1 x LightCycler 480 Probes Master (Roche), 0.5 µM of each primer and 0.125 µM of each fluorescent probe. Template was 0.5 µl gDNA. PCR conditions for the reaction were an initial 5 minutes DNA denaturation step at 95^o^C followed by 45 cycles of 10 seconds at 94^o^C, and 20 seconds at 53^o^C before a final cooling at 40^o^C for 10 seconds. Data obtained through the *TP53* specific reactions were normalized by adjusting for B2M. These normalized values were divided by the corresponding values from a reference sample (pooled DNA from >10 healthy donors). Samples were considered to have reduced copy number if the sample / reference ratio was <0.75, and increased copy number if the ratio was >1.25.

**Primers:**

*TP53* LOH1:

Forward: GAAGACCCAGGTCCAGATGA

Reverse: CTGCCCTGGTAGGTTTTCTG

Probe: 6FAM-CCTCCTGGCCCCTGTCATCT-BBQ

*TP53* LOH2:

Forward: CAGTCTACCTCCCGCCATAA

Reverse: CCACAACAAAACACCAGTGC

Probe: 6FAM-TGTGCGTCAGAAGCACCCAG-BBQ

*B2M*:

Forward: CATCCAGCAGAGAATGGAAAG

Reverse: GAAAGACCAGTCCTTGCTGAA

Probe: Cy-5-TGGGTTTCATCCATCCGACA-BBQ
